# Loss of exosomal micro-RNA-200b-3p from hypoxia cancer-associated fibroblasts reduces sensitivity to 5-flourouracil in colorectal cancer through targeting high-mobility group box 3

**DOI:** 10.3389/fonc.2022.920131

**Published:** 2022-10-05

**Authors:** Hang Yuan, Bingchen Chen, Rui Chai, Wenjing Gong, Ziang Wan, Boan Zheng, Xinye Hu, Yang Guo, Shan Gao, Qiaoqiong Dai, Peng Yu, Shiliang Tu

**Affiliations:** General Surgery, Cancer Center, Department of Colorectal Surgery, Zhejiang Provincial People’s Hospital (Affiliated People’s Hospital, Hangzhou Medical College), Hangzhou, China

**Keywords:** miR-200b-3p directly targets HMGB3 CAFs, exosome, hypoxia, miR-200b-3p, HMGB3

## Abstract

Hypoxia-mediated tumor progression is a major problem in colorectal cancer (CRC). MicroRNA (miR)-200b-3p can attenuate tumorigenesis in CRC, while exosomal miRNAs derived from cancer-associated fibroblasts (CAFs) can promote cancer progression. Nevertheless, the function of exosomal miR-200b-3p derived from CAFs in CRC remains unclear. In this study, CAFs and normal fibroblasts (NFs) were isolated from CRC and adjacent normal tissues. Next, exosomes were isolated from the supernatants of CAFs cultured under normoxia and hypoxia. Cell viability was tested using the cell counting kit-8 assay, and flow cytometry was used to assess cell apoptosis. Cell invasion and migration were evaluated using the transwell assay. Dual-luciferase was used to investigate the relationship between miR-200b-3p and high-mobility group box 3 (HMBG3). Reverse transcription-quantitative polymerase chain reaction (RT-qPCR) was performed to determine the miR-200b-3p and HMBG3 level. Our results found that the miR-200b-3p level was sharply reduced in CRC tissues compared to adjacent normal tissues. Additionally, the miR-200b-3p level was reduced in exosomes derived from hypoxic CAFs compared to exosomes derived from CAFs under normoxia. Exosomes derived from hypoxic CAFs weakened the sensitivity of CRC cells to 5-fluorouracil (5-FU) compared to hypoxic CAFs-derived exosomes. However, hypoxic CAFs-derived exosomes with upregulated miR-200b-3p increased the sensitivity of CRC cells to 5-fluorouracil (5-FU) compared to hypoxic CAFs-derived exosomes. In addition, HMBG3 was identified as the downstream target of miR-200b-3p in CRC cells, and its overexpression partially reversed the anti-tumor effect of the miR-200b-3p agomir on CRC *via* the mediation of the β-catenin/c-Myc axis. Furthermore, compared to exosomes derived from normoxia CAFs, exosomes derived from hypoxic CAFs weakened the therapeutic effects of 5-FU on CRC *in vivo via* the upregulation of HMGB3 levels. Collectively, the loss of exosomal miR-200b-3p in hypoxia CAFs reduced the sensitivity to 5-FU in CRC by targeting HMGB3. Thus, our research outlines a novel method for the treatment of CRC.

## Introduction

Colorectal cancer (CRC) is the third most common malignancy worldwide, exhibiting high incidence and mortality rates (1, 2). Although surgical resection and adjuvant therapies have been used to treat CRC, the overall 5-year survival rate of patients with CRC remains low (3–5). For CRC, 5-fluorouracil (5-FU) is used as a chemotherapeutic agent; however, drug resistance caused by its long-term use decreases the clinical efficacy of 5-FU (6, 7). Therefore, it is imperative to increase the sensitivity of CRC cells to 5-FU for effective treatment.

A hypoxic tumor microenvironment (TME) is associated with tumor progression and chemoresistance (8–10). Hypoxia can contribute to aggressive tumor behavior and induce distant metastasis (11, 12). Cancer-associated fibroblasts (CAFs) are key players in the TME, which could promote chemoresistance during cancer progression (13–15). Hypoxic cancer cells can promote CAF activation, which, in turn, leads to increased invasion and stemness of tumor cells (16). Meanwhile, hypoxic CAFs can promote endothelial cell angiogenesis, facilitating the metastasis of malignant tumors (17). Thus, hypoxic CAFs may play vital roles in chemoresistance and metastasis during tumor progression.

Crosstalk between CAFs and tumor cells is often mediated by exosomes (18). Exosomes (40–100 nm) contain different nucleic acids including miRNAs that can modulate the communication between CAFs and cancer cells (19, 20). It has been shown that CAF-secreted exosomal miR−181d−5p could inhibit CRC cell sensitivity to 5-FU (21). Chen et al. suggested that exosomal miR-590-3p from CAFs could confer radioresistance to CRC cells (22). Furthermore, the miR-200b-3p level was reduced in CRC, and it can mitigate oxaliplatin resistance by targeting tubulin beta 3 class III (23). Nevertheless, whether exosomal miR-200b-3p derived from CAFs could affect the sensitivity of CRC cells to 5-FU remains unclear.

In this study, we aimed to investigate the function of exosomal miR-200b-3p derived from CAFs in CRC. We hope that our research provides new strategies for increasing the sensitivity of CRC cells to 5-FU.

## Materials and methods

### Ethics statement

This study was approved by the Ethics Committee of the Zhejiang Provincial People’s Hospital (No. 20211009ZPPH).

### Tissue collection

Five pairs of CRC and adjacent normal tissues were collected from patients with CRC who had undergone surgery at the Zhejiang Provincial People’s Hospital. Primary human CAFs were isolated from tumor tissues, and NFs were extracted from the adjacent normal tissues (24). This study was approved by the Ethics Committee of the Zhejiang Provincial People’s Hospital. Isolated CAFs and NFs were identified by fibroblast activation protein (FAP) staining, as previously described (25).

### Cell culture and transfection

CAFs and NFs were maintained in Dulbecco’s minimal essential medium/Ham’s F12 (DMEM/F12) containing fetal bovine serum (FBS; 10%) at 37°C. For hypoxic stimulation, CAFs were maintained in 1% O_2_ for 24 h. Human CRC cells (SW480 and SW620) were purchased from the American Type Culture Collection and cultured in the DMEM medium (Invitrogen) containing FBS (10%) in the presence of 5% CO_2_ at 37°C.

The miR-200b-3p agomir and negative control (NC) were purchased from RIBOBIO (Guangzhou, China). Before hypoxic stimulation, CAFs were transfected with the NC or miR-200b-3p agomir for 48 h using Lipofectamine 2000 (Invitrogen).

In addition, pcDNA3.1-HMGB3 and pcDNA3.1 control were purchased from RIBOBIO. CAFs were transfected with pcDNA3.1-HMGB3 or pcDNA3.1 control for 48 h.

### RT-qPCR assay

TRIpure Reagent (ELK Biotechnology) was used to extract the RNA from cells, and the EntiLink 1st Strand cDNA Synthesis Kit (ELK Biotechnology) was used to reverse-transcribe RNA into cDNA. Next, qPCR was conducted on the StepOne Real-Time PCR System using the EnTurbo SYBR Green PCR SuperMix Kit (ELK Biotechnology). U6 served as an internal control for miR-200b-3p. β-actin served as an internal control for HMGB3. The 2^−ΔΔCq^ method was used for the quantification of data. U6, forward 5′-CTCGCTTCGGCAGCACAT-3′, reverse 5′-AACGCTTCACGAATTTGCGT-3′; miR-200b-3p, forward 5′-GGCCCTAATACTGCCTGGTA-3′, reverse 5′-CTCAACTGGTGTCGTGGAGTC-3′; β-actin, forward 5′-GTCCACCGCAAATGCTTCTA-3′, reverse 5′-TGCTGTCACCTTCACCGTTC-3′; HMGB3, forward 5′-CTATGATCGGGAAATGAAGGATTAT-3′, reverse 5′-TTCTCATACTTCTCCTTCAGCTTTG-3′.

### Western blotting

BCA kit was used to determine the protein concentration. Proteins were then electrophoresed on sodium dodecyl sulfate-polyacrylamide gels (10%) and transferred to a polyvinylidene fluoride membrane. Primary antibodies against HMGB3 (1:1000; Proteintech Group), CD63 (1:5000; Proteintech Group), tumor susceptibility 101 (TSG101; 1:2000; Proteintech Group), β-catenin (1:2000; Proteintech Group), c-Myc (1:2000; Proteintech Group), non-phospho (active) β-Catenin (1:1000; Abcam), and β-actin (1:1000; Proteintech Group) were used to incubate the membranes overnight at 4°C. Then, the membranes were incubated with the appropriate secondary antibodies for 1 h. An ECL kit was used for protein detection.

### Cell apoptosis detection

CRC cells were trypsinized and resuspended. Next, 5 μl of Annexin V and 5 μl of propidium iodide were applied to stain the cells in the dark for 15 min. Flow cytometry (BD Biosciences, Franklin Lake, NJ, USA) was used to analyze the apoptotic cells. FlowJo (v10.6.2; BD Biosciences) was used to quantify the data.

### Trypan blue staining

First, 90 µl of the cell suspension was transferred to a cryovial. Trypan blue staining buffer (0.4%, 10 μl) was mixed with the cell suspension and incubated for 5 min. Finally, the number of dead cells (stained blue) were counted and observed under a conventional light microscope.

### Exosome isolation and identification

CAFs were cultured under both normoxic (CAFs-N) and hypoxic (CAFs-H) conditions. Exosomes were purified from CAFs-N (CAFs-N-Exo) or CAFs-H (CAFs-H-Exo) *via* ultracentrifugation, as previously described (26). Exosomes were then resuspended and stored immediately at −80°C. For NTA, the size distribution of exosomes was determined using a ZetaView nanoparticle tracking analyzer (Particle Metrix). For transmission electron microscopy (TEM), the exosomes were fixed, loaded, and stained with 1% phosphotungstic acid. The morphology of the exosomes was then observed using a transmission electron microscope.

### Exosome uptake

PKH26 dye (Sigma) was used to label CAFs-N- and CAFs-H-derived exosomes (CAFs-N-Exo and CAFs-H-Exo, respectively) for 30 min. Later on, the labeled exosomes (red) were co-cultured with SW480 cells at 37°C. Subsequently, fluorescence microscopy was used to observe the labeled exosomes.

### Cell counting kit (CCK)-8 assay

SW480 cells (5000 cells/well) were seeded onto a 96-well plate overnight. After that, each well was exposed to a CCK-8 reagent (10 μl; Beyotime). After 2 h of incubation, the absorbance at 450 nm was assessed using a microplate reader.

### Co-culture system

CAFs-N or CAFs-H cells were seeded into Transwell polyester permeable supports. SW480 cells were seeded into the bottom chamber. SW480 cells (bottom chamber) were co-cultured with transfected CAFs-N or CAFs-H cells (upper chamber) for 48 h. Subsequently, a CCK-8 assay was used to determine cell viability.

### Transwell assays

Cell migration and invasion assays were performed using Transwell assays in 24-well Transwell chambers (Corning). SW480 cells suspended in 100 µl of serum-free DMEM were plated onto the upper chamber, and 500 μl of DMEM plus 10% FBS was added to the lower chamber. After 24 h of incubation, the cells that passed through the filter were stained with 0.2% crystal violet. Subsequently, cells were observed under a microscope. For the invasion assay, the lower chamber was pre-coated with Matrigel (BD Biosciences).

### Luciferase reporter assay

The wild-type or mutant 3′-untranslated region (UTR) of HMGB3 was inserted into the pGL6-miR‐based luciferase reporter vector (Beyotime). SW480 cells were co-transfected with HMGB3 wild-type or mutant plasmids and the miR-200b-3p agomir or agomir NC using Lipofectamine 2000 for 48 h. Next, the luciferase activity was detected with the Dual Luciferase Reporter Assay System.

### Animal study

Four-week-old BALB/c nude mice were purchased from Weitonglihua, China. SW480 cells (10^7^ cells) were injected into the left flank of nude mice subcutaneously. When the tumors reached approximately 200 mm^3^ in size, the animals were divided randomly into four groups: control, 5-FU, 5-FU + CAFs-N/NC-Exo, and 5-FU + CAFs-H/NC-Exo. Animals were injected with 5-FU (25 mg/kg) intraperitoneally once a week. Meanwhile, PBS, CAFs-N/NC-Exo, or CAFs-H/NC-Exo was injected intratumorally twice weekly for 3 weeks. The tumor volume was measured every week using the following formula: volume = (length × width^2^)/2. The mice were sacrificed on day 21 and the tumors were removed. Subsequently, the tumor volumes and tumor weights were measured. Cell apoptosis was assessed using the APO-BrdU TUNEL assay kit. The animal study was approved by the Ethics Committee of the Zhejiang Provincial People’s Hospital.

### Statistical analysis

Data are expressed as the mean ± standard deviation. Group comparisons were performed using one-way analysis of variance and Tukey’s test. Differences were considered statistically significant at p < 0.05. All data were analyzed in triplicate.

## Results

### MiR-200b-3p can be transferred from hypoxic CAFs to SW480 cells *via* exosomes

**Figure 1:**
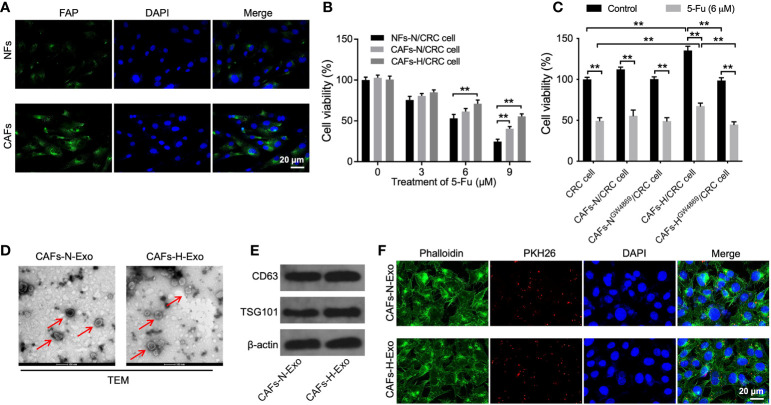
Hypoxic CAFs-derived exosomes can be transferred to SW480 cells. **(A)** NFs and CAFs were isolated from CRC tissues and adjacent normal tissues, respectively. IF staining for FAP expression in CAFs and NFs. **(B)** CRC cells were treated with 0, 3, 6, or 9 μM 5-FU for 48 h. Then, CRC cells were co-cultured with NFs-N, CAFs-N, or CAFs-H. The viability of CRC cells was tested using the CCK8 assay. **(C)** CRC cells were treated with 6 μM 5-FU for 48 h. Then, CRC cells were co-cultured with CAFs-N, CAFs-H, or GW4869-treated CAFs-N or CAFs-H. The viability of CRC cells was tested by CCK8 assay. **(D)** Identification of exosomes (CAFs-N-Exo and CAFs-H-Exo) derived from CAFs cultured under normoxia and hypoxia by TEM. Red arrow points indicate exosomes. **(E)** Western blot analysis of exosome surface markers CD63 and TSG101 in CAFs-N-Exo and CAFs-H-Exo. **(F)** Fluorescence images showed the internalization of PKH26-labeled CAFs-N-Exo or CAFs-H-Exo (red) by SW480 cells. **p < 0.01.

Tumor cell growth is closely related to the TME, in which hypoxia and CAFs are two major factors (27, 28). Thus, CAFs and NFs were isolated from the CRC and adjacent normal tissues, respectively. As shown in **Figure 1A**, the level of FAP (a specific fibroblast marker) was much higher in CAFs than in NFs, indicating the successful isolation of CAFs. To investigate whether hypoxic CAFs could affect 5-FU sensitivity in CRC cells, SW480 cells were co-cultured with CAFs-N or CAFs-H. As shown in **Figures 1B, C**, 5-FU sharply reduced CRC cell viability, whereas CAFs-H notably reversed that change, suggesting that CAFs-H significantly reduced the sensitivity of CRC cells to 5-FU.

It has been shown that exosomes carrying miRNAs could modulate the communication between CAFs and cancer cells (19, 20). Thus, to explore whether hypoxic CAFs-derived exosomes result in this effect, exosome inhibitor GW4869 was used (29). As indicated in **Figure 1C**, reducing the exosome secretion of hypoxic CAFs by GW4869 inhibited the ability of hypoxic CAFs to decrease sensitivity to 5-FU in CRC cells. These results showed that exosomes play a key role in mediating the communication between hypoxic CAFs and SW480 cells.

Next, CAFs-N-Exo and CAFs-H-Exo were isolated from the supernatants of CAFs-N or CAFs-H, respectively. These two vesicles were identified as round and cup-shaped membrane-coated particles using TEM (**Figure 1D**). In addition, these two vesicles expressed the exosomal markers CD63 and TSG101 (**Figure 1E**). Meanwhile, PKH26-labeled exosomes were observed in SW480 cells (**Figure 1F**), suggesting that exosomes could be absorbed by SW480 cells.

As shown in **Supplementary Figure 1A**, the miR-200b-3p level was significantly downregulated in CRC tissues. Additionally, the miR-200b-3p level in SW480 cells was markedly upregulated by the miR-200b-3p agomir (**Supplementary Figure 1B**). Meanwhile, compared to exosomes derived from miRNA NC-transfected normoxia CAFs (CAFs-N/NC-Exo), the miR-200b-3p level was significantly downregulated in exosomes derived from miRNA NC-transfected hypoxic CAFs (CAFs-H/NC-Exo) (**Supplementary Figure 1C**). Furthermore, miR-200b-3p levels in CRC cells were notably elevated by exosomes derived from normoxia or hypoxic CAFs that were transfected with the miR-200b-3p agomir (CAFs-N/miR-200b-3p agomir-Exo or CAFs-H/miR-200b-3p agomir-Exo) (**Supplementary Figure 1D**). Thus, miR-200b-3p can be transferred from hypoxic CAFs to SW480 cells *via* exosomes.

### Exosomal miR-200b-3p derived from hypoxic CAFs increases the sensitivity of CRC cells to 5-FU

**Figure 2:**
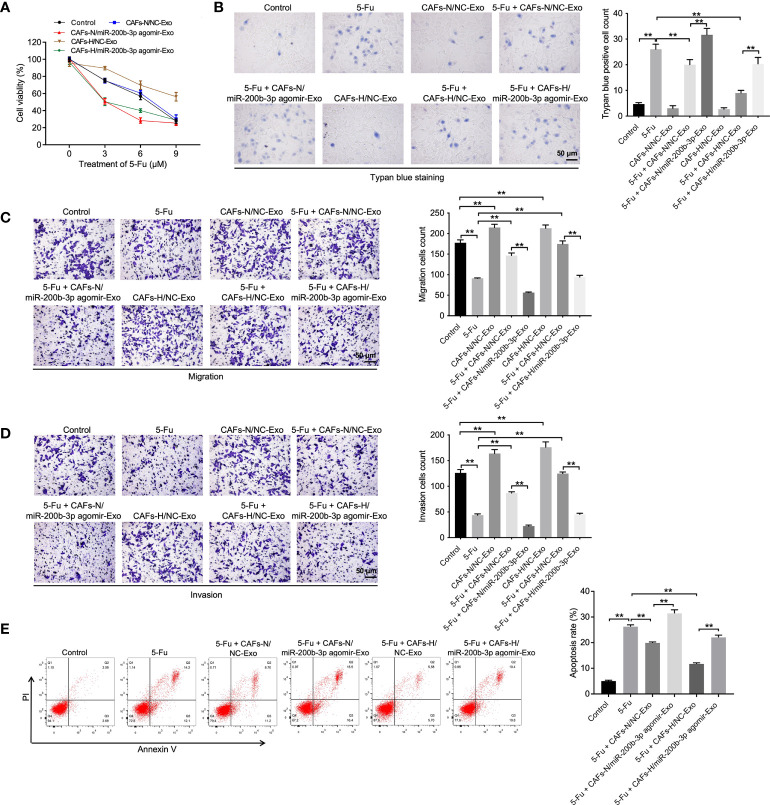
Exosomal miR-200b-3p derived from hypoxic CAFs increases the sensitivity of CRC cells to 5-FU. CRC cells were treated with 3, 6, or 9 μM 5-FU for 48 h. Then, CRC cells were treated with CAFs-N/NC-Exo, CAFs-N/miR-200b-3p agomir-Exo, CAFs-H/NC-Exo, or CAFs-H/miR-200b-3p agomir-Exo. **(A)** The viability of CRC cells was tested using the CCK8 assay. **(B)** The death of CRC cells was tested using trypan blue staining. **(C, D)** The migration and invasion of CRC cells was detected using the transwell assay. **(E)** The apoptosis of CRC cells was tested by flow cytometry. **p < 0.01.

To investigate the function of hypoxic CAFs-derived exosomal miR-200b-3p in CRC, CCK-8, trypan blue staining, transwell, and flow cytometry assays were performed. As indicated in **Figures 2A–E**, 5-FU treatment remarkably reduced CRC cell viability, migration, and invasion and promoted cell death and apoptosis, whereas CAFs-N/NC-Exo or CAFs-H/NC-Exo significantly reversed these phenomena, suggesting that normoxia or hypoxic CAFs could reduce the sensitivity of CRC cells to 5-FU. Significantly, compared to CAFs-H/NC-Exo, CAFs-H/miR-200b-3p agomir-Exo reduced CRC cell viability, migration, and invasion and increased cell death (**Figures 2A–E**). In summary, exosomal miR-200b-3p derived from hypoxic CAFs could increase the sensitivity of CRC cells to 5-FU.

### HMGB3 is the target mRNA of miR-200b-3p

**Figure 3:**
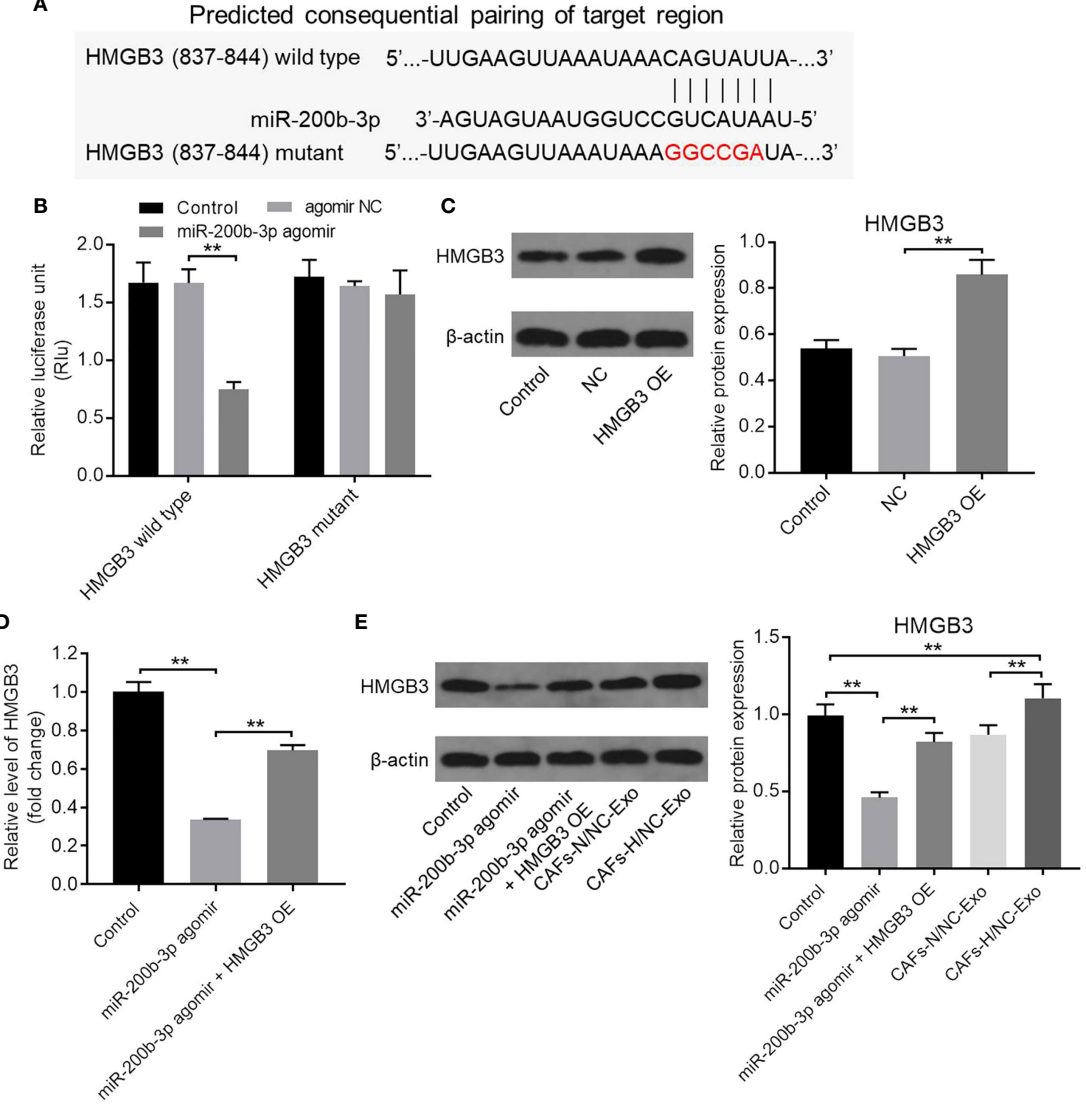
HMGB3 was identified to be the target mRNA of miR-200b-3p. **(A)** Targetscan was used to predict the downstream mRNA of miR-200b-3p. **(B)** The relative luciferase activity in WT/MT-HMGB3 was tested using the dual luciferase report assay. **(C)** SW480 cells were transfected with NC or pcDNA3.1-HMGB3 for 48 h. The HMGB3 level in SW480 cells was investigated by Western blot. The relative expression was quantified by normalizing to β-actin. **(D)** The HMGB3 level in CRC cells was evaluated using RT-qPCR. **(E)** SW480 cells were treated with the miR-200b-3p agomir, miR-200b-3p agomir + HMGB3 OE, CAFs-N/NC-Exo or CAFs-H/NC-Exo for 48 h. The level of HMGB3 in CRC cells was investigated by Western blot. **P < 0.01.

TargetScan (https://www.targetscan.org/vert_71/) was used to identify the downstream targets of miR-200b-3p. As shown in **Figure 3A**, the 3′UTR of HMGB3 mRNA contained a complementary miR-200b-3p binding sequence, suggesting that HMGB3 might be a downstream target of miR-200b-3p. In addition, a dual luciferase reporter assay was performed to validate whether HMGB3 is indeed a target of miR-200b-3p. As indicated in **Figure 3B**, the relative luciferase activity in WT-HMGB3 was significantly reduced by the miR-200b-3p agomir. Moreover, compared with the NC group, the HMGB3 level was significantly upregulated in SW480 cells transfected with pcDNA3.1-HMGB3 (**Figure 3C**). Meanwhile, the HMGB3 level in CRC cells was significantly inhibited by the miR-200b-3p agomir, while this effect was reversed by HMGB3 overexpression (**Figures 3D, E**). Furthermore, CAFs-H/NC-Exo markedly increased the HMGB3 level in CRC cells compared to the control group (**Figure 3E**). Taken together, HMGB3 is a direct binding target mRNA of miR-200b-3p.

### Overexpression of miR-200b-3p increases the sensitivity of CRC cells to 5-FU by inhibiting HMGB3

**Figure 4:**
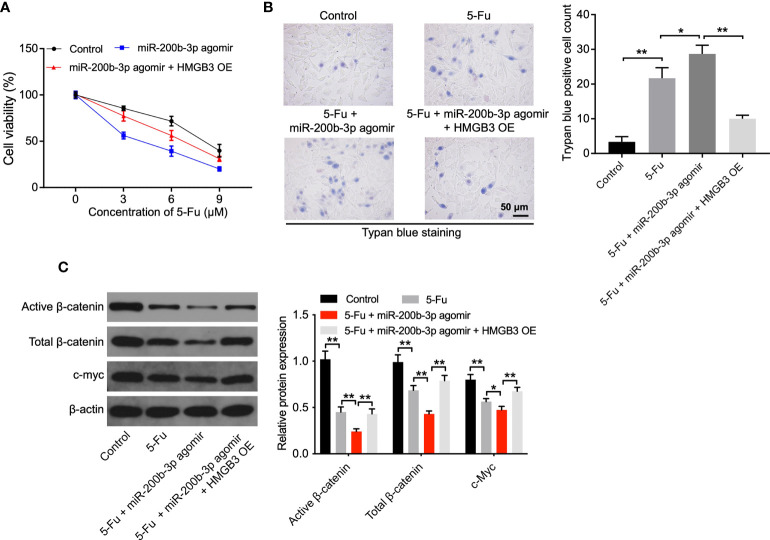
The miR-200b-3p agomir increases the sensitivity of CRC cells to 5-FU *via* inhibiting HMGB3. **(A)** SW480 cells were treated with 0, 3, 6, or 9 μM 5-FU for 48 h. Then, cells were treated with the miR-200b-3p agomir or miR-200b-3p agomir + HMGB3 OE. The viability of SW480 cells was tested by CCK8 assay. **(B)** The death of SW480 cells was tested using trypan blue staining. **(C)** The expressions of active β-catenin, total β-catenin, and c-Myc in SW480 cells were detected by Western blot. *p < 0.05, **p < 0.01.

To investigate the mechanism by which the miR-200b-3p agomir increases the sensitivity of CRC cells to 5-FU, CCK-8, and trypan blue staining assays were performed. As shown in **Figures 4A, B** and **Supplementary Figure 2**, the anti-tumor effects of 5-FU in CRC cells were enhanced by the miR-200b-3p agomir; however, these changes were notably reversed by HMGB3 overexpression. In addition, the level of active β-catenin, total β-catenin, and c-Myc in SW480 cells was notably decreased by 5-FU (**Figure 4C**). As expected, the miR-200b-3p agomir further reduced the active and total β-catenin level and c-Myc level in SW480 cells compared with the 5-FU group (**Figure 4C**). However, the effects of miR-200b-3p on these proteins were notably abolished by HMGB3 overexpression (**Figure 4C**). These results indicated that miR-200b-3p overexpression could increase the sensitivity of CRC cells to 5-FU by inhibiting HMGB3/β-catenin/c-Myc signaling.

### Loss of miR-200b-3p in hypoxic CAFs-derived exosomes weakened the anti-tumor effects of 5-FU on CRC cells *in vivo*

**Figure 5:**
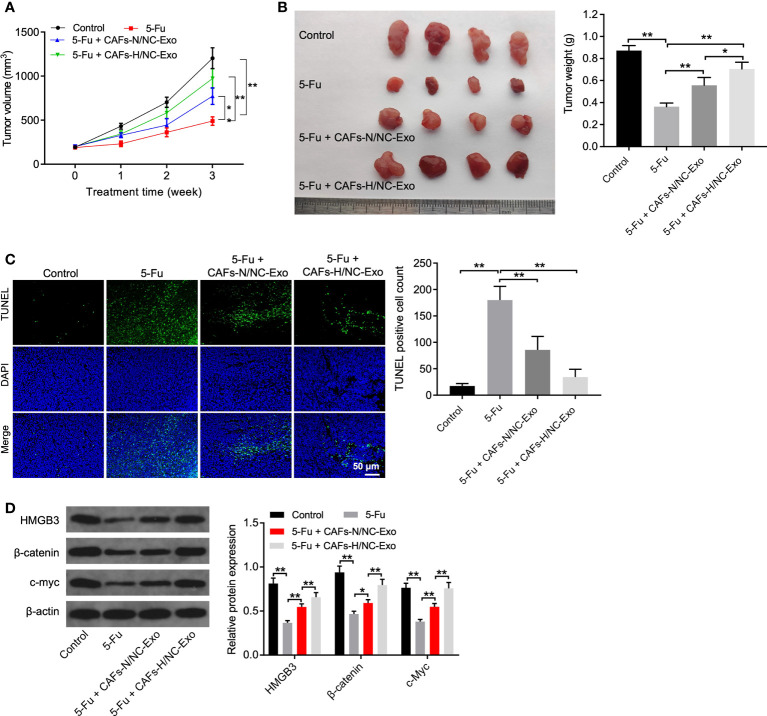
Loss of miR-200b-3p in hypoxic CAFs-derived exosomes weakened the anti-tumor effects of 5-FU on CRC *in vivo*. **(A, B)** Tumor volume and tumor weight of xenograft tumors. **(C)** TUNEL analysis of cell apoptosis in tumor tissues. **(D)** Western blot analysis of HMGB3, β-catenin, and c-Myc expressions in tumor tissues. *p < 0.05, **p < 0.01.

Finally, we investigated the effect of CAFs-H-Exo on the tumorigenicity of CRC *in vivo*. As shown in **Figures 5A, B**, 5-FU treatment obviously reduced the tumor volume and tumor weight, whereas CAFs-N/NC-Exo or CAFs-H/NC-Exo treatment partially reversed these changes. In addition, CAFs-H/NC-Exo notably reduced the apoptotic effect of 5-FU on tumor tissues (**Figure 5C**). Moreover, 5-FU significantly downregulated the HMGB3, c-Myc, and β-catenin level in tumor tissues; however, these changes were reversed by CAFs-N/NC-Exo or CAFs-H-Exo (**Figure 5D**). Meanwhile, compared with the CAFs-N/NC-Exo group, CAFs-H/NC-Exo further weakened the anti-tumor effects of 5-FU on CRC *in vivo* (**Figures 5A–D**). Significantly, the miR-200b-3p level was significantly downregulated in CAFs-H/NC-Exo, compared to CAFs-N/NC-Exo (Supplementary **Figure 1C**). These data show that the loss of miR-200b-3p in hypoxic CAFs-derived exosomes could reduce the sensitivity of SW480 cells to 5-FU *in vivo via* upregulating HMGB3/β-catenin/c-Myc signaling.

## Discussion

Hypoxia plays a crucial role in tumor progression (30). CAFs can regulate cancer progression, acting as key modulators in the TME (31, 32). Exosomes are important mediators of cell-to-cell crosstalk between tumor and surrounding cells *via* transmitting miRNAs (33). Jin et al. suggested that CAF-derived exosomal miR-3656 could promote the progression and development of esophageal squamous cell carcinoma (34), and Fan et al. suggested that exosomal miR-26a from CAFs could facilitate endometrial cancer progression by targeting the signal transducer and activator of the transcription 3/chitinase 3-like 1 signaling pathway (35). However, the specific role of hypoxia in CAFs requires further exploration. Thus, we explored the crosstalk between hypoxic CAFs and CRC cells. Our results showed that compared with hypoxic CAF-derived exosomes, hypoxic CAF-derived exosomes with the miR-200b-3p agomir can increase the sensitivity of CRC cells to 5-FU, and this is the first study to explore the function of exosomal miR-200b-3p in CRC. In contrast, Chen et al. found that exosomal miR-590-3p derived from CAFs was able to confer radioresistance to CRC cells (22), and Yin et al. suggested that CAF-derived exosomes with upregulated miR-135b-5p levels can promote CRC cell growth by inhibiting thioredoxin-interacting protein (36). These results showed that miRNAs involved in the communication between hypoxic CAFs and CRC cells.

Exosome-mediated transfer of miRNAs from CAFs to cancer cells can induce chemoresistance during cancer progression (33, 37). Exosomal miR-200b was found to act as a biomarker in human cancers including CRC (38–40). In addition, the miR-200b level was found to be downregulated in CRC (41). Thus, we investigated the role of exosomal miR-200b-3p in CRC. In our study, compared to normoxia CAFs-derived exosomes, the miR-200b-3p level was significantly downregulated in hypoxic CAF-derived exosomes. Meanwhile, CAFs-H/NC-Exo weakened the anti-tumor effects of 5-FU on CRC *in vitro and in vivo*, compared with the CAFs-N/NC-Exo group. These results showed that compared to normoxia CAFs-derived exosomes, loss of exosomal miR-200b-3p in hypoxia CAFs could reduce the sensitivity of CRC cells to 5-FU. Additionally, miR-200b-3p can be transferred from hypoxic CAFs to CRC cells *via* exosomes. Normoxia or hypoxic CAF-derived exosomes reduced the sensitivity of CRC cells to 5-FU, while these effects were reversed by normoxia or hypoxic CAF-derived exosomes with upregulated miR-200b-3p levels, respectively. These data showed that increasing the exosomal miR-200b-3p level derived from hypoxic CAFs could reverse the effect of hypoxic CAF-derived exosomes on 5-FU sensitivity in CRC cells.

HMGB3 acts as the downstream mRNA of miR-200b-3p in CRC. HMGB3 is a crucial mediator of cancer development and associated with chemoresistance during cancer progression (42, 43). Yu et al. found that lncRNA OIP5-AS1 could facilitate the chemoresistance of breast cancer cells to trastuzumab *via* the upregulation of HMGB3 (43). Additionally, HMGB3 could facilitate CRC progression *via* activating WNT/β-catenin/c-Myc signaling (44). β-catenin is a vital modulator of Wnt signaling and plays important roles in cancer metastasis and chemoresistance (45, 46). Meanwhile, c-Myc is a downstream target of β-catenin, which could contribute to chemoresistance in CRC (47–49). In this study, we found that hypoxic CAFs-derived exosomes could increase the HMGB3 level in CRC cells. Meanwhile, hypoxic CAFs-derived exosomes could reverse 5-FU-induced HMGB3, c-Myc, and β-catenin downregulation in tumor tissues, suggesting that hypoxic CAFs-derived exosomes could reduce the sensitivity of CRC cells to 5-FU *via* activating HMGB3/β-catenin/c-Myc signaling. In addition, the miR-200b-3p agomir could increase the sensitivity of 5-FU to CRC cells by the inhibition of HMGB3/β-catenin/c-Myc signaling. Collectively, exosomes with upregulated miR-200b-3p levels derived from hypoxic CAFs could increase the sensitivity of 5-FU to CRC cells *via* inhibiting the HMGB3/β-catenin/c-Myc axis.

This study has some limitations. First, other target mRNAs of miR-200b-3p in CRC were not explored. One miRNA has been found to target multiple genes (50). Evidence has shown that miR-200b could target fascin actin-bundling protein 1 (FSCN1), and p70S6K1 and these two target genes (FSCN1 and p70S6K1) are involved in the chemoresistance of cancers (51, 52). Thus, further studies are needed to investigate whether exosomal miR-200b-3p could modulate CRC progression *via* targeting other genes (e.g. FSCN1 and p70S6K1). Second, other signaling pathways involved in exosomal miR-200b-3p-mediated CRC progression could not be investigated. Future studies should focus on addressing these limitations for more novel insights.

## Conclusion

Our results revealed that the loss of exosomal miR-200b-3p in hypoxia CAFs could reduce the sensitivity of CRC cells to 5-FU by upregulating HMGB3. Thus, our research may be beneficial for future studies aiming at the development of possible therapeutic strategies for CRC.

## Data availability statement

The original contributions presented in the study are included in the article/**Supplementary Materials**. Further inquiries can be directed to the corresponding author.

## Ethics statement

The studies involving human participants were reviewed and approved by the ethics committee of Zhejiang Provincial People’s Hospital (No. 20211009ZPPH). The patients/participants provided their written informed consent to participate in this study. The animal study was reviewed and approved by the ethics committee of Zhejiang Provincial People’s Hospital.

## Author contributions

The study was conceived and designed by HY, BC, RC, and ST, and the experiments were performed by WG, ZW, and BZ. Data were analyzed, and the paper was written by XH, YG, SG, QD, and PY. All authors have reviewed and agreed to the submission of the manuscript.

## Funding

This study was financially supported by the Natural Science Foundation of Zhejiang Province (No. LY18H160041, LY17H160064, and LQ21H160042), the Funding Project of Health and Family Planning Commission of Zhejiang Province (2018KY217), and the Funding Project Administration of Traditional Chinese Medicine of Zhejiang Province (2018ZA009).

## Conflict of interest

The authors declare that the research was conducted in the absence of any commercial or financial relationships that could be construed as a potential conflict of interest.

## Publisher’s note

All claims expressed in this article are solely those of the authors and do not necessarily represent those of their affiliated organizations, or those of the publisher, the editors and the reviewers. Any product that may be evaluated in this article, or claim that may be made by its manufacturer, is not guaranteed or endorsed by the publisher.

## Supplementary material

Supplementary Figure 1 MiR-200b-3p was downregulated in CRC tissues. **(A)** The level of miR-200b-3p in CRC tissues or adjacent normal tissues was tested by RT-qPCR. **(B)** CAFs cells were transfected with the NC or miR-200b-3p agomir. The level of miR-200b-3p in CAFs was investigated by RT-qPCR. **(C)** The level of miR-200b-3p in CAFs-N/NC-Exo and CAFs-H/NC-Exo was evaluated by RT-qPCR. **(D)** CRC cells were treated with CAFs-N/NC-Exo, CAFs-N/miR-200b-3p agomir-Exo, CAFs-H/NC-Exo, or CAFs-H/miR-200b-3p agomir-Exo. The level of miR-200b-2p in CRC cells was evaluated by RT-qPCR. **p < 0.01.

Supplementary Figure 2 The miR-200b-3p agomir increases the sensitivity of SW620 cells to 5-FU. SW620 cells were treated with 6 μM 5-FU for 48 h. Then, cells were treated with the miR-200b-3p agomir or miR-200b-3p agomir + HMGB3 OE. The viability of SW620 cells was tested by CCK8 assay.
