# Supplementary figures and images for: Loss of exosomal micro-RNA-200b-3p from hypoxia cancer-associated fibroblasts reduces sensitivity to 5-flourouracil in colorectal cancer through targeting high-mobility group box 3
Source: Front Oncol. 2022 Oct 5;12:920131. doi: 10.3389/fonc.2022.920131 (PMC9581251; doi:10.3389/fonc.2022.920131)

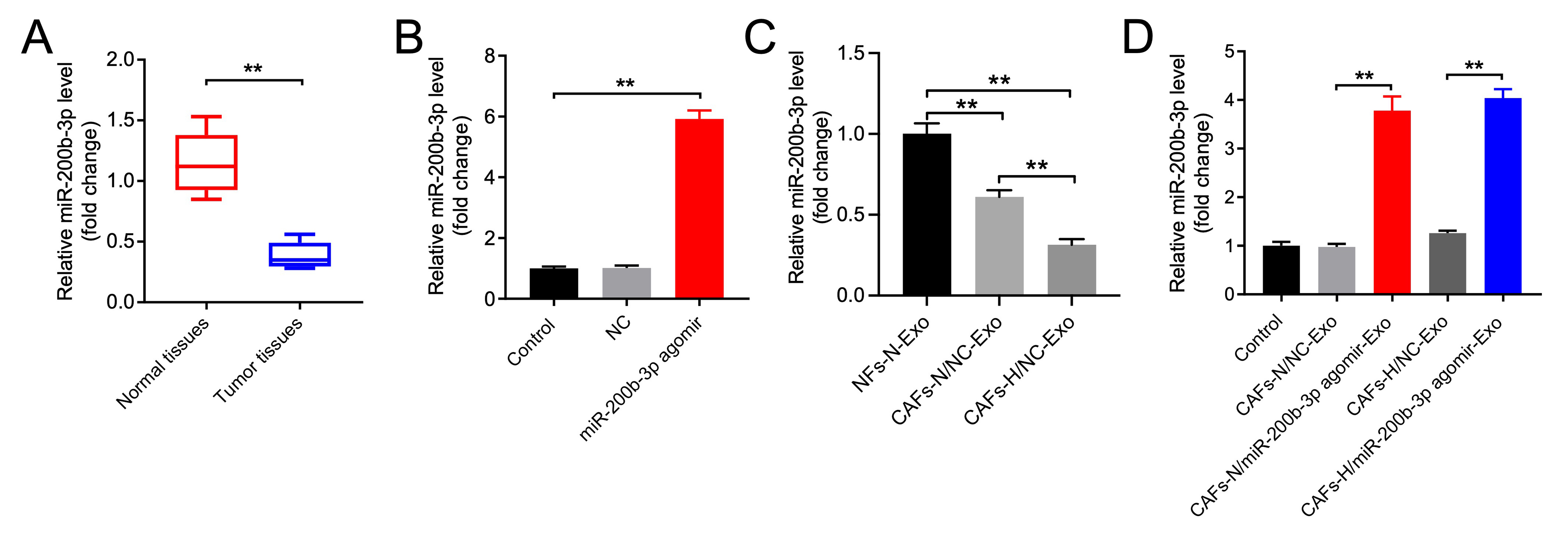

Supplement: Supplementary Figure 1 — MiR-200b-3p was downregulated in CRC tissues. (A) The level of miR-200b-3p in CRC tissues or adjacent normal tissues was tested by RT-qPCR. (B) CAFs cells were transfected with the NC or miR-200b-3p agomir. The level of miR-200b-3p in CAFs was investigated by RT-qPCR. (C) The level of miR-200b-3p in CAFs-N/NC-Exo and CAFs-H/NC-Exo was evaluated by RT-qPCR. (D) CRC cells were treated with CAFs-N/NC-Exo, CAFs-N/miR-200b-3p agomir-Exo, CAFs-H/NC-Exo, or CAFs-H/miR-200b-3p agomir-Exo. The level of miR-200b-2p in CRC cells was evaluated by RT-qPCR. **p < 0.01. [file Image_1.jpeg]

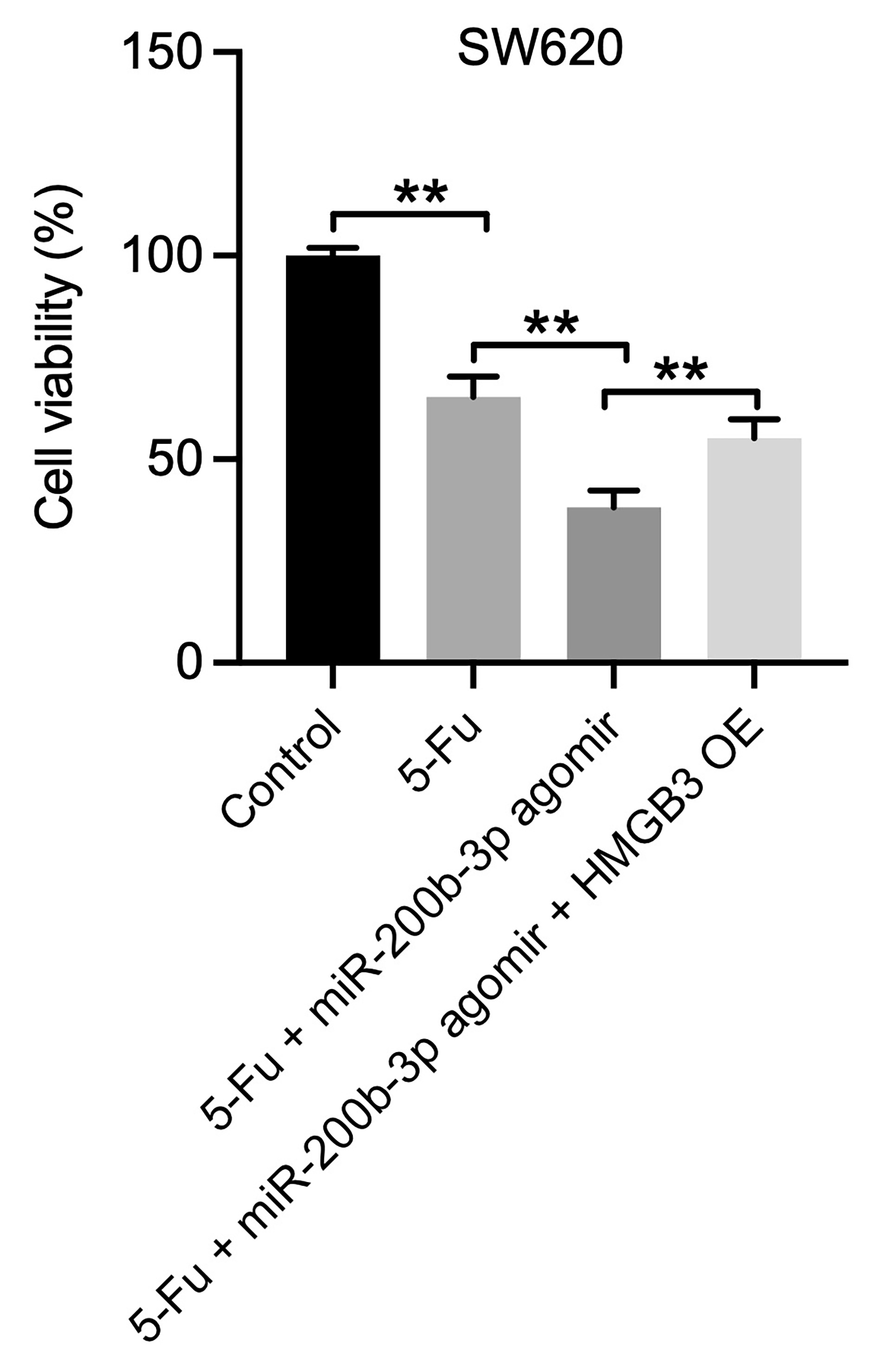

Supplement: Supplementary Figure 2 — The miR-200b-3p agomir increases the sensitivity of SW620 cells to 5-FU. SW620 cells were treated with 6 μM 5-FU for 48 h. Then, cells were treated with the miR-200b-3p agomir or miR-200b-3p agomir + HMGB3 OE. The viability of SW620 cells was tested by CCK8 assay. [file Image_2.jpeg]
